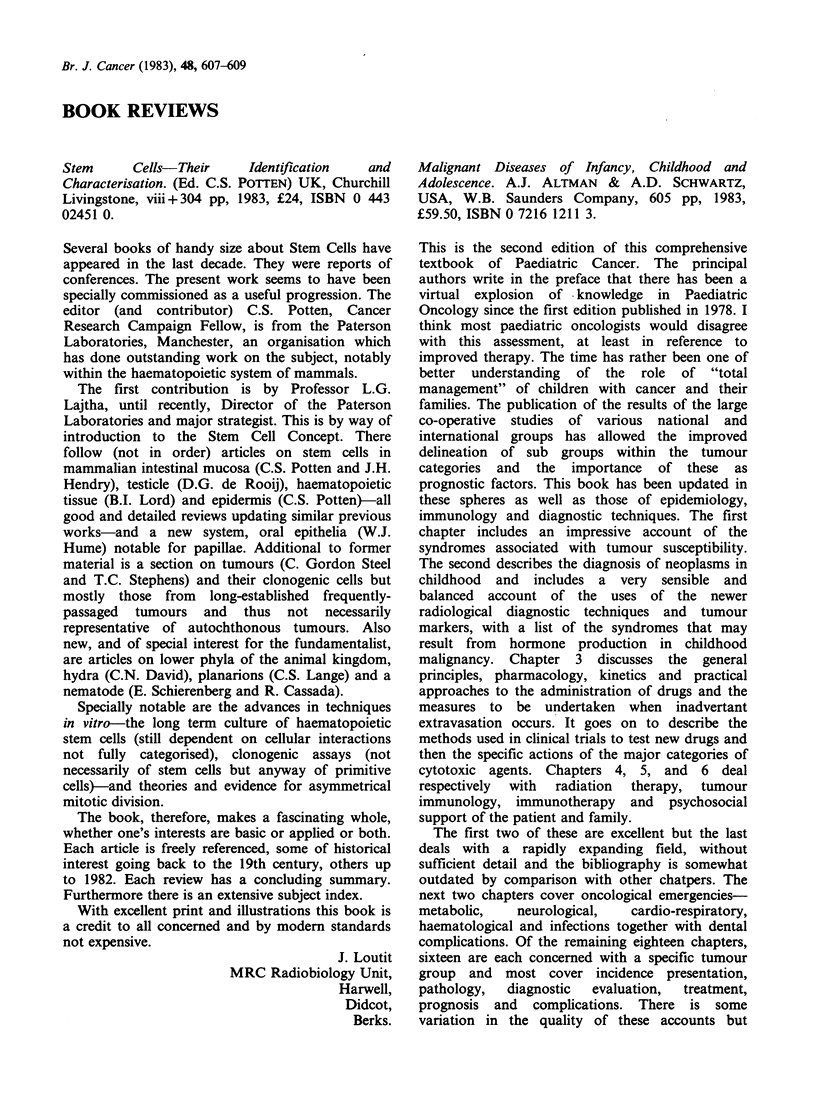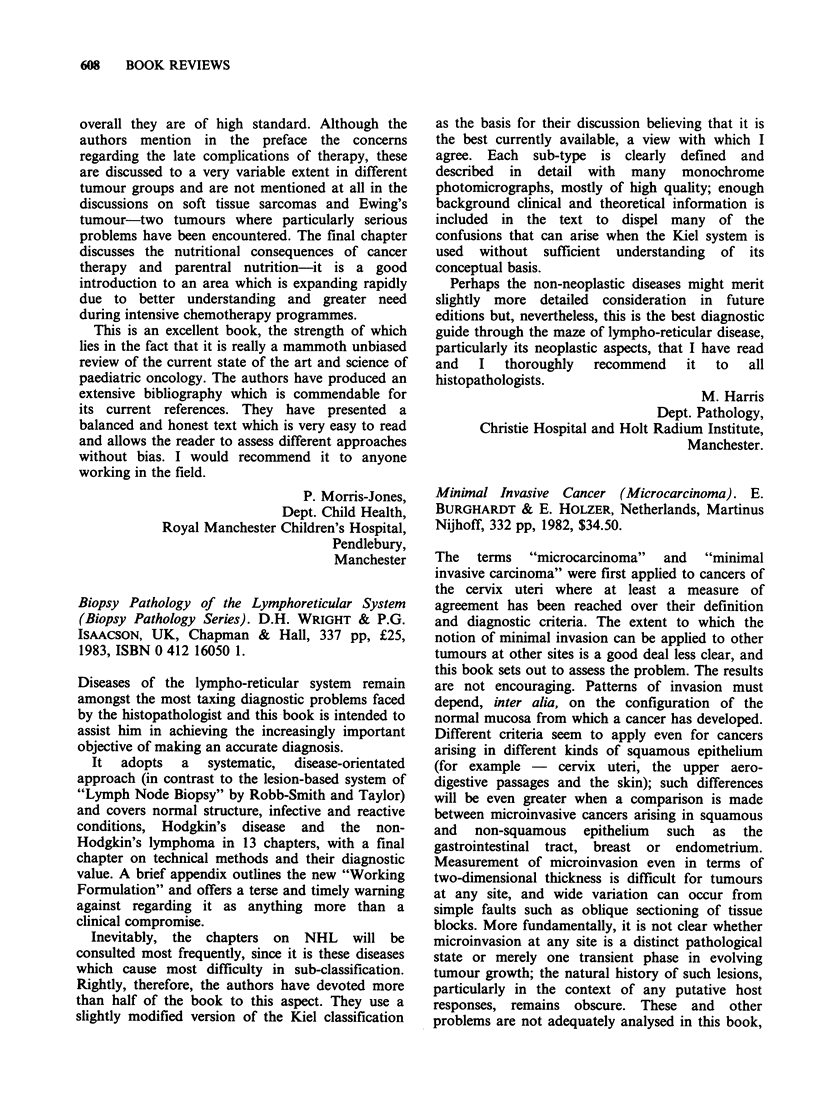# Malignant Diseases of Infancy, Childhood and Adolescence

**Published:** 1983-10

**Authors:** P. Morris-Jones


*Malignant Diseases of Infancy, Childhood and Adolescence.* A.J. ALTMAN & A.D. SCHWARTZ, USA, W.B. Saunders Company, 605 pp, 1983, £59.50, ISBN 0 7216 1211 3.

This is the second edition of this comprehensive textbook of Paediatric Cancer. The principal authors write in the preface that there has been a virtual explosion of knowledge in Paediatric Oncology since the first edition published in 1978. I think most paediatric oncologists would disagree with this assessment, at least in reference to improved therapy. The time has rather been one of better understanding of the role of "total management" of children with cancer and their families. The publication of the results of the large co-operative studies of various national and international groups has allowed the improved delineation of sub groups within the tumour categories and the importance of these as prognostic factors. This book has been updated in these spheres as well as those of epidemiology, immunology and diagnostic techniques. The first chapter includes an impressive account of the syndromes associated with tumour susceptibility. The second describes the diagnosis of neoplasms in childhood and includes a very sensible and balanced account of the uses of the newer radiological diagnostic techniques and tumour markers, with a list of the syndromes that may result from hormone production in childhood malignancy. Chapter 3 discusses the general principles, pharmacology, kinetics and practical approaches to the administration of drugs and the measures to be undertaken when inadvertant extravasation occurs. It goes on to describe the methods used in clinical trials to test new drugs and then the specific actions of the major categories of cytotoxic agents. Chapters 4, 5, and 6 deal respectively with radiation therapy, tumour immunology, immunotherapy and psychosocial support of the patient and family.

The first two of these are excellent but the last deals with a rapidly expanding field, without sufficient detail and the bibliography is somewhat outdated by comparison with other chatpers. The next two chapters cover oncological emergencies—metabolic, neurological, cardio-respiratory, haematological and infections together with dental complications. Of the remaining eighteen chapters, sixteen are each concerned with a specific tumour group and most cover incidence presentation, pathology, diagnostic evaluation, treatment, prognosis and complications. There is some variation in the quality of these accounts but overall they are of high standard. Although the authors mention in the preface the concerns regarding the late complications of therapy, these are discussed to a very variable extent in different tumour groups and are not mentioned at all in the discussions on soft tissue sarcomas and Ewing's tumour—two tumours where particularly serious problems have been encountered. The final chapter discusses the nutritional consequences of cancer therapy and parentral nutrition—it is a good introduction to an area which is expanding rapidly due to better understanding and greater need during intensive chemotherapy programmes.

This is an excellent book, the strength of which lies in the fact that it is really a mammoth unbiased review of the current state of the art and science of paediatric oncology. The authors have produced an extensive bibliography which is commendable for its current references. They have presented a balanced and honest text which is very easy to read and allows the reader to assess different approaches without bias. I would recommend it to anyone working in the field.

P. Morris-Jones,
Dept. Child Health,
Royal Manchester Children's Hospital,
Pendlebury,
Manchester